# Removal of heavy metals from aqueous solution using platinum nanopartcles/Zeolite-4A

**DOI:** 10.1186/2052-336X-12-7

**Published:** 2014-01-07

**Authors:** Sofia Mehdizadeh, Sodeh Sadjadi, Seyed Javad Ahmadi, Mohammad Outokesh

**Affiliations:** 1 Department of Energy Engineering, Sharif University of Technology, Tehran, Iran; 2 Nuclear fuel cycle school, Nuclear Science and Technology Research Institute, Tehran, Iran

**Keywords:** Platinum nanopartcles, Zeolite-4A, Heavy metals, Adsorption

## Abstract

The effects of varying operating conditions on metals removal from aqueous solution using a novel platinum nanopartcles/Zeolite-4A adsorbent are reported in this paper. Characterization of the adsorbent showed successful production of platinum nanopartcles on Zeolite-4A using 3 Wt% platinum. The effects of operation conditions on metals removal using this adsorbent were investigated. The optimal metals adsorption was observed at pH 7, 0.1 g/10 mL dosage and 30 min contact time. Sorption data have been interpreted in terms of Langmuir and Freundlich isotherms.

## Background

Water contaminated by heavy metal ions had become much more serious with a rapid development of industries and competitive use of fresh water in many parts of the world [1]. Heavy metals are not biodegradable and tend to accumulate in living organisms, causing various diseases and disorders [2-4].Therefore, the removal of heavy metal ions from water has become an important subject today.

Several conventional methods exist for the removal of heavy metal pollutants from wastewater. These methods include precipitation, electroplating, chemical coagulation, ion-exchange, membrane separation, and electro kinetics. However, most of these methods have limitations, which include high cost, unavailability, and generation of large volumes of secondary waste and poor removal efficiency [5-7]. Considering from the economy and efficiency point of view, adsorption is regarded as the most promising and widely used method among all these [8]. The efficiency of adsorption depends on many factors, including the surface area, pore size distribution, polarity, and functional groups of the adsorbent [9]. One of the main limitations of the sorption techniques is the massive mass transport resistance due to the size of the adsorbents. To overcome this limitation, the use of new adsorbents has attracted considerable attention in recent years.

The application of nanotechnology to the purification and treatment of wastewater may potentially revolutionize water treatment processes because of their unusual physical and chemical properties owing to their extremely small size and large specific surface area. Due to these characteristics, nanomaterials have found wide applications in adsorption and the removal of heavy metal ions from aqueous solutions [10].

Zeolites are microporous crystalline solids with well-defined structures, which have unique ion exchange and sorption properties, and are widely used in a large number of water treatment processes [11].

The objective of the present study is to investigate the adsorption potential of Pt on zeolite in the removal of heavy metal ions from aqueous solution. The effects of pH, adsorbent dosage, contact time and temperature on adsorption capacity of Pt/Zeolite have been investigated. The Langmuir and Freundlich isotherms models are applied to the sorption data to calculate the different parameters and the best fittings achieved.

## Methods

### Material and instrument

The particle size of the nanocrystalline particle was examined using transmission electron microscopy (TEM, Philips EM208S at 100KV). The X-ray diffraction (XRD) pattern of the catalyst powders was provided by a (Philips PW 1800) diffractometer. To determine the concentration of component, Inductively Coupled Plasma (ICP, Perkin Elmer 5500) was used. Different pH of Samples was measured with pH meter (Schott CG-841) and centrifuge (Beckman J-21C device) was applied for separating solid particles from the solution. The Shaker bath (Infors AG) was used for the stirring system at constant temperature to determine the distribution coefficients.

Zeolite4A were obtained from Pars zeolite Co. (Tehran, Iran) and Hexachloroplatinic acid: H_2_PtCl_6_.6H_2_O (99.9%, metals basis) and Poly vinylpyrrolidone (PVP, Mw= 29 000) was purchased from Merck All the compounds used to prepare the reagent solutions were of analytic reagent grade. The heavy metal ions solutions containing 50, 100, 250 and 500 mg/L each of La^3+^, C0^2+^, Ba^2+^ and Ni^2+^ ions were prepared by dissolving a weighed quantity of the respective nitrate salts in distilled water.

### Synthesis of the platinum nanoparticle

The synthesis procedure was started by adding 133 mg PVP into a mixture of 20 cm^3^ of 6.0 mM H_2_PtCl_6_.6H_2_O aqueous solution and 180 cm^3^ pure ethanol. The mixture then was refluxed for 3 h. Afterward, the solvent was evaporated, and the obtained black precipitate was thoroughly washed with water and ethanol, and dried at ambient condition.

### Preparation of Pt/Zeolite

About 20 cm^3^ of H_2_PtCl_6_.6H_2_O aqueous solution (6 mM) was mixed with 133 mg PVP and 180 cm^3^ of ethanol. The mixture was quickly added to 1.5 g of the zeolite 4A, and refluxed for 3 h. The solvent then was evaporated and the obtained granules were calcined at 450°C for 12 h in a stream of muffle furnace.

### Batch adsorption experiments

Batch adsorption experiment was conducted for removal of studied heavy metal at temperature (25±0.1°C). The 0.1 g of pure zeolite 4A and Pt/zeolite 4A was left in contact with 10 ml of heavy metal ion solutions (100 mg/L). Samples were collected at 5, 10, 15, 30, 60, and 120 min to determine optimal shaking time. The pH values were adjusted to 3.5, 4.5, 5.5, 6.5 and 7 using 0.2 M CH_3_COOH and CH_3_COONa solution. The effect of adsorbent dosage was studied with the adsorbent dose of 0.25, 0.05, 0.1, 0.2, and 0.3 g per 10 ml of test solution (100 mg/L). The adsorption studies were also carried out at 5, 25, 40, and 60°C to determine the effect of temperature. During the adsorption process, the test bottles were agitated on a shaker at 200 rpm. At the end of the experiment, samples were withdrawn from the test bottles and filtered by centrifuge and residual metal ion concentration was measured by using inductively coupled plasma-atomic emission spectroscopy (ICP). The equilibrium sorption capacity was determined from Eq. (1):

(1) $$\mathrm{qe}=\left(\mathrm{Ci}-\mathrm{Ce}\right)\frac{V}{M}$$

Where *C*i (mg/L) is the initial concentration, *C*e (mg/L) the concentration at equilibrium, *q*e (mg/mg) the amount of metal ions adsorbed at equilibrium, *m* (g) the adsorbent mass and *V* (L) is the solution volume. Removal efficiency of metal ions by the adsorbent is considered in percentage as Eq. (2):

(2) $$\text{Removal}\;\text{efficiency}=\frac{\left(\mathrm{Ci}-\mathrm{Ce}\right)}{\mathrm{Ci}}\times 100$$

Where Ci and Ce are the initial and final metal ion concentrations, respectively.

## Results and discussion

### Preparation and characterization of adsorbent

Monodisperse platinum particles of 20–30 nm were synthesized by modified alcohol reduction methods according to the literature [12]. Methanol served as solvent for dissolving Pt salts and PVP, and as a reducing agent of Pt according to the following reaction:

$$H_{2}\mathrm{PtC}l_{6}+{2\mathrm{CH}}_{3}\mathrm{OH}\to Pt^{0}+{2\mathrm{CH}}_{2}O+6\mathrm{HCl}$$

4A with a pore diameter of 9.0 nm was used as a support due to its high surface area and ordered mesoporous structure. Platinum/zeolite adsorbent was prepared as discussed in section 2. The loading of the platinum on the zeolite sample was measured by dissolving the sample in aqua regia, and analyzing of the Pt content of the supernatant by ICP method. The loading was found to be around 3 Wt%.

**Figure 1:**
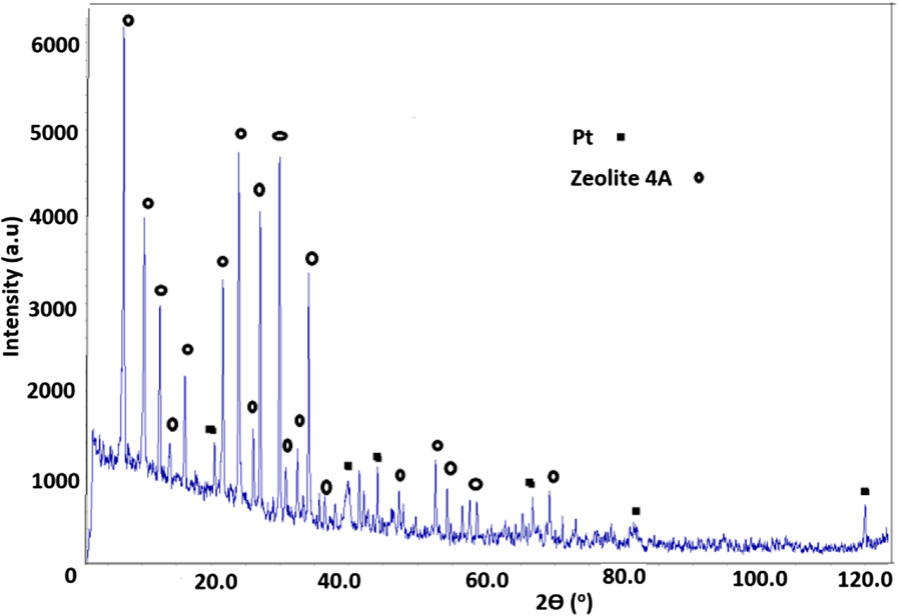
XRD patterns of Pt particles on Zeolite 4A.

**Figure 2:**
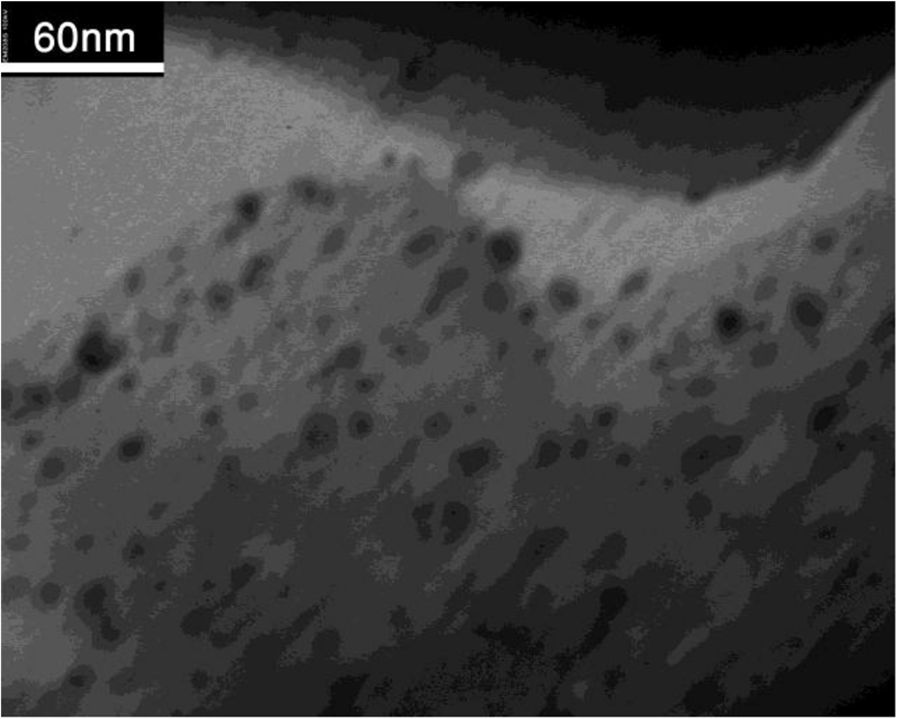
Transmission electron microscopy of Pt/Zeolite 4A.

This adsorbent was characterized by XRD (Figure 1) and TEM (Figure 2) measurements. The Pt reflections are seen in the XRD patterns of zeolite 4A (Figure 1). TEM image of Pt/zeolite 4A (Figure 2) show that the particles are well-dispersed in the entire channel structures.

### Adsorption studies

The prepared Pt/zeolite 4A was then used as adsorbent in the removal of toxic metal ions in aqueous solution. The removal efficiency (R%) of the metal ions on this adsorbent (Table 1) show good affinity of this material for a number of ions such as Co (II), Ba (II), La (III) and Ni (II) ions in water. In order to confirm the utility of platinum nanoparticles in this adsorbent, the compression of efficiency of Pt/zeolite 4A with zeolite 4A has been carried out. The results are shown in Table 1. It is clear that Pt/ zeolite 4A has the better adsorption than zeolite 4A.

**Table 1:** The removal efficiency (R%) of the metal ions on Pt/zeolite 4A with zeolite 4A

**Compounds**	**R% Zeolite**	**R% Pt/Zeolite**
Hg(II)	58.5062	98.9612
La(III)	65.1290	99.9890
Sr(II)	87.8493	99.3774
Pb(II)	68.9862	79.0831
Ni(II)	78.8953	99.9888
U(VI)	55.9448	68.2650
Ba(II)	78.3013	99.9888
Th(IV)	46.2192	59.4667
Zr(IV)	69.8656	79.8844
Co(II)	78.8202	99.9897
Mo(VI)	48.8550	59.3219

To optimize the adsorption system, the effects of various parameters such as adsorbent dose, pH, and time on the adsorption of Co (II), Ba (II), La (III) and Ni (II) ions were studied.

### Effect of adsorbent dosage

**Figure 3:**
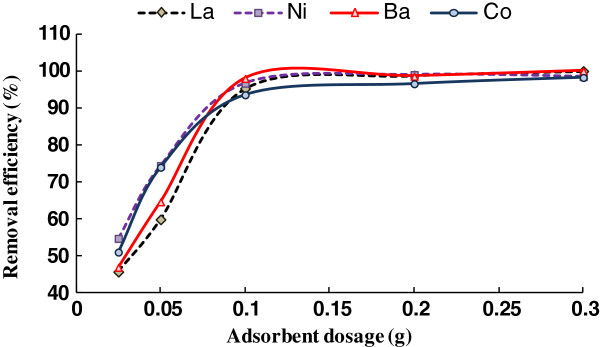
Effect of adsorbent dosage.

Adsorbent dosage is one of the important parameters in adsorption processes because it determines the capacity of an adsorbent for a given initial concentration of the adsorbate under a given set of operating conditions. To achieve this aim, a series of batch experiments were conducted with the adsorbent dose of 0.25, 0.05, 0.1, 0.2, and 0.3 g per 10 ml of test solution. Figure 3 shows the effect of adsorbent dosage on the adsorption of Co (II), Ba (II), La (III) and Ni (II) ions. When the addition of the adsorbent dose increased, the percentage removal of metal ions also increased. A maximum removal of 98% of Co (II) and Ni (II) and 99% of Ba (II) and La (III) ions respectively was obtained at 0.1 g of the adsorbent. It can be seen from Figure 3 that an adsorbent dose of 0.1 g is sufficient for optimal removal of metal ions in aqueous solutions. A further increase in the quantity of adsorbent dose will not have any significant effect on the removal of metal ions from the solution. The initial increment in adsorption capacity with increase in adsorbent dosage was expected because as the adsorbent dose increases the number of adsorbent particles increases, thus more surface areas were available for metals attachment [13].

### Effect of contact time

**Figure 4:**
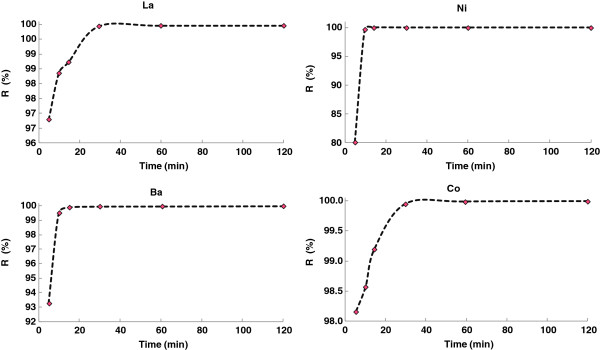
Effect of contact time.

The result of the effect of contact time on removal efficiency for Co (II), Ba (II), La (III) and Ni (II) using 0.1 g of Pt/zeolite 4A at room temperature (25±0.1°C) is represented in Figure 4. The adsorption rate was observed as rapid in the first 20 min, followed by a gradual increase with time until equilibrium adsorption was noticed at 30 min. The fast adsorption at the initial stage was probably due to the initial concentration gradient between the adsorbate in solution and the number of vacant sites available on the adsorbent surface. The attainment of equilibrium adsorption might have been due to reduction in the available active adsorption sites on the adsorbent with time resulting to limited mass transfer of the adsorbate molecules from the bulk liquid to the external surface of adsorbent.

### Effect of pH on adsorption

**Figure 5:**
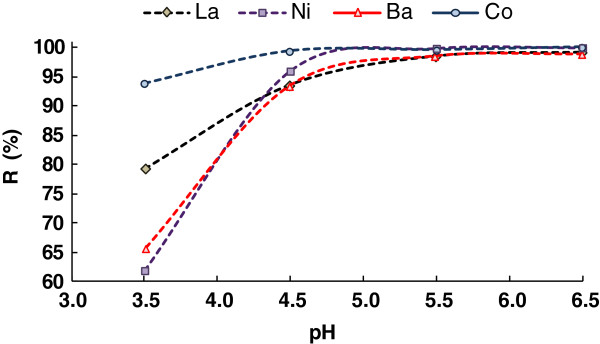
Effect of pH value on removal of heavy metal ions.

The pH of the solution had been reported to be important factor in adsorption processes. The variations in adsorption capacity of Pt/zeolite 4A with increasing pH is shown in Figure 5, it could be inferred that the adsorption capacity increased as solution pH increased from pH 3.5 to 7 and capacity decreases above pH 7.

At low pH the concentration of protons was high and metal binding sites became positively charged repelling the Co (II), Ba (II), La (III) and Ni (II) cations. With an increase in pH, the negative charge density on the adsorbent increases due to deprotonation of the metal binding sites, thus increasing metal adsorption. The maximum adsorption observed in the pH 7 and the subsequent reduction in adsorption capacity was probably due the partial hydrolysis of metal ions. Furthermore, the low solubility of hydrolyzed metals species would have resulted into precipitation of metals at pH above 7, thereby reducing adsorption capacity of Pt/zeolite 4A.

### Adsorption isotherms

The adsorption isotherms are very important in describing the adsorption behavior of solutes on the specific adsorbents. In this work, two important isotherm models such as Langmuir and Freundlich were selected and studied.

Langmuir sorption isotherm models the monolayer coverage of the sorption surfaces and assumes that sorption occurs on a structurally homogeneous adsorbent and all the sorption sites are energetically identical. The Freundlich expression is an empirical equation based on a heterogeneous surface [14]. The general form of Freundlich and Langmuir isotherm is as follows. Langmuir isotherm (Eq. (3)):

(3) $$\frac{C_{e}}{q_{e}}=\frac{1}{Q^{0}b}+\frac{1}{Q^{0}}C_{e}$$

Freundlich isotherm (Eq. (4)):

(4) $${\text{Logq}}_{e}={\text{LogK}}_{f}+\frac{1}{n}{\text{LogC}}_{e}$$

where *qe* (in mg/mg) is the adsorbate amount adsorbed by 1 g of adsorbent, *Ce* (in mg/L) is the equilibrium concentration of adsorbate in the solution, *Q*^o^ is the monolayer adsorption capacity (mg/mg), *b* is the constant related to the adsorption intensity, *K*_f_ is constant indicative of the relative sorption capacity of Pt/zeolite 4A (mg/g) and 1/*n* is the constant indicative of the intensity of the sorption process.

**Figure 6:**
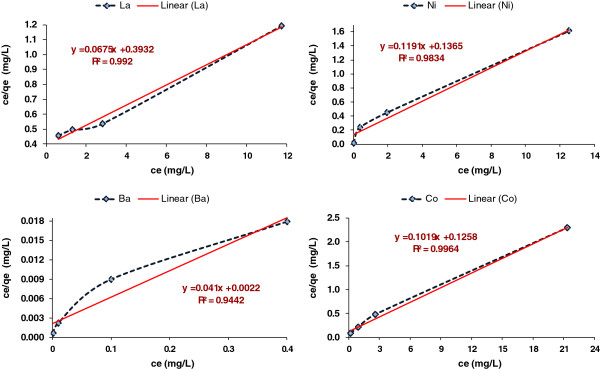
Langmuir isotherm for Co (II), Ba (II), La (III) and Ni (II) adsorption on Pt/zeolite 4A.

**Figure 7:**
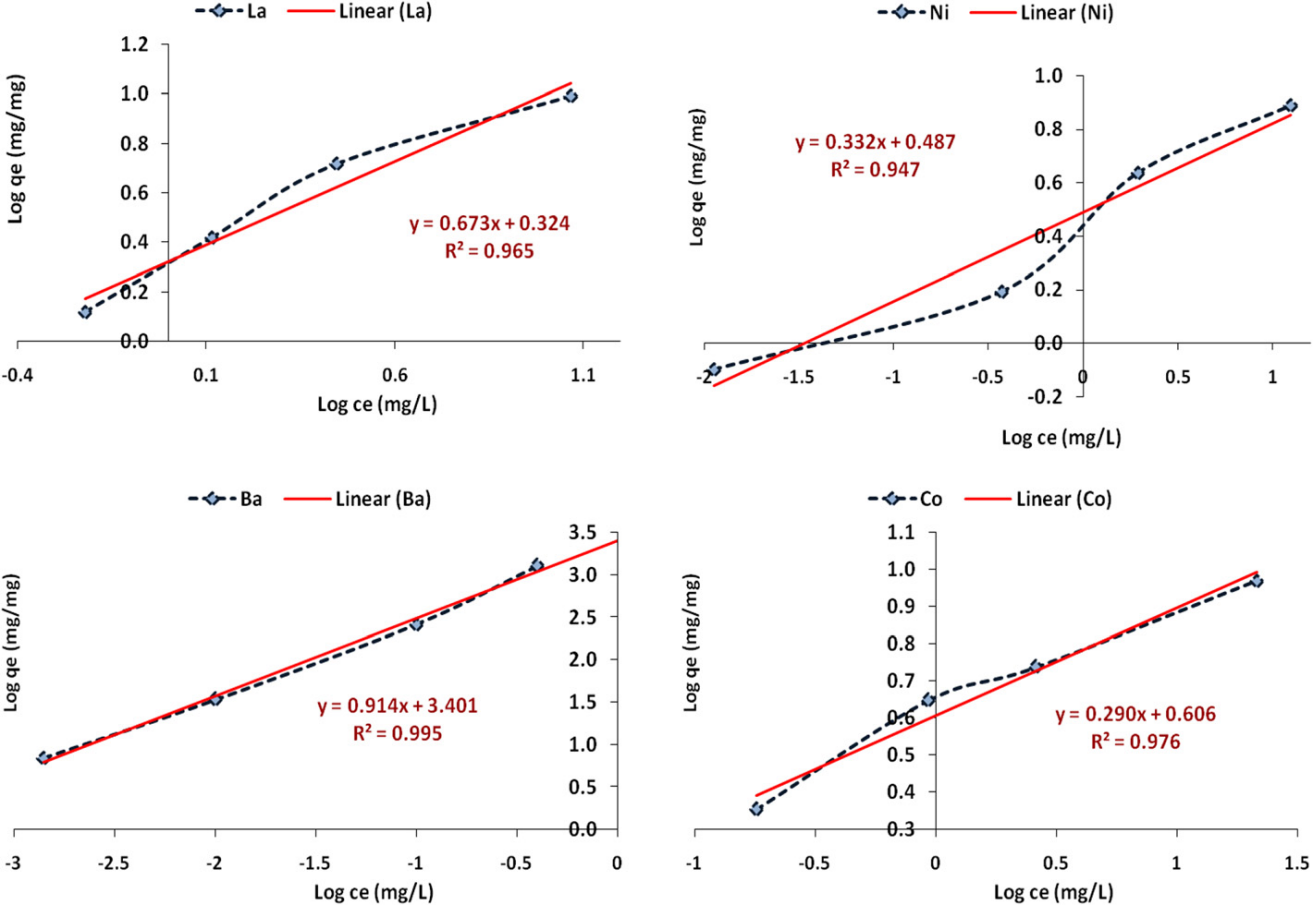
Freundlich isotherm for Co (II), Ba (II), La (III) and Ni (II) adsorption on Pt/zeolite 4A.

The values of Langmuir and Freundlich parameters for the removal of Co (II), Ba (II), La (III) and Ni (II) metals ions are presented in Table 2 and the pictorial illustrations are shown in Figures 6 and 7.

**Table 2:** Isothermal adsorption models for the adsorption of Co (II), Ba (II), La (III) and Ni (II) adsorption on Pt/zeolite 4A

**Metal ion**	**Langmuir**			**Freundlich**		
	**Q**^**o **^**(mg/g)**	**b (L/mg)**	***R***^***2***^	***n***	***K***_***f ***_**(mg/g)**	***R***^***2***^
La (III)	14.81	0.172	0.992	1.485	2.113	0.9657
Co(II)	0.981	0.810	0.9964	3.446	4.044	0.9761
Ni (II)	0.839	0.875	0.9834	3.008	3.075	0.9474
Ba (II)	24.39	18.64	0.9442	2.519	30.005	0.9953

It was observed that results fitted better in the Freundlich model in terms of the correlation factor (R^2^) value, recording 0.99 for Ba (II) and the higher Langmuir correlation factor (R^2^) values of 0.996, 0.992 and 0.983 for the adsorption Co (II), La (III) and Ni (II) respectively, strongly suggests that the Langmuir model gives a better fit to the experimental data and so the nature of adsorption of metal ions on the adsorbent is more compatible with Langmuir assumptions.

### Adsorption kinetics

**Figure 8:**
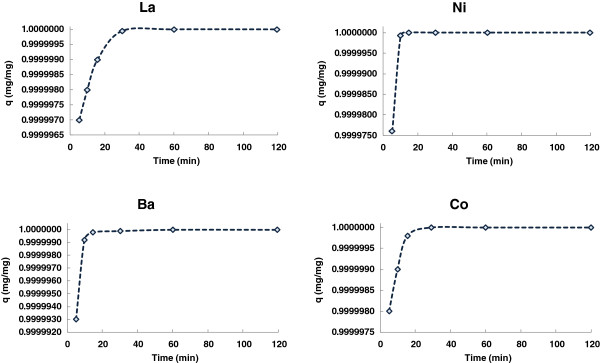
Adsorption kinetics of Co (II), Ba (II), La (III) and Ni (II) ions onto Pt/zeolite 4A.

The adsorption of the Co (II), Ba (II), La (III) and Ni (II) ions onto Pt/zeolite 4A as a function of contact time was investigated and data were given in Figure 8. The experiment was carried with the initial concentrations of 100 mg/L whereby 0.1 g of adsorbent was contacted with 10 mL of Co (II), Ba (II), La (III) and Ni (II) (II) aqueous solution. Adsorption was rapid in the first stages and then slowed considerably as the reaction approached equilibrium.

**Figure 9:**
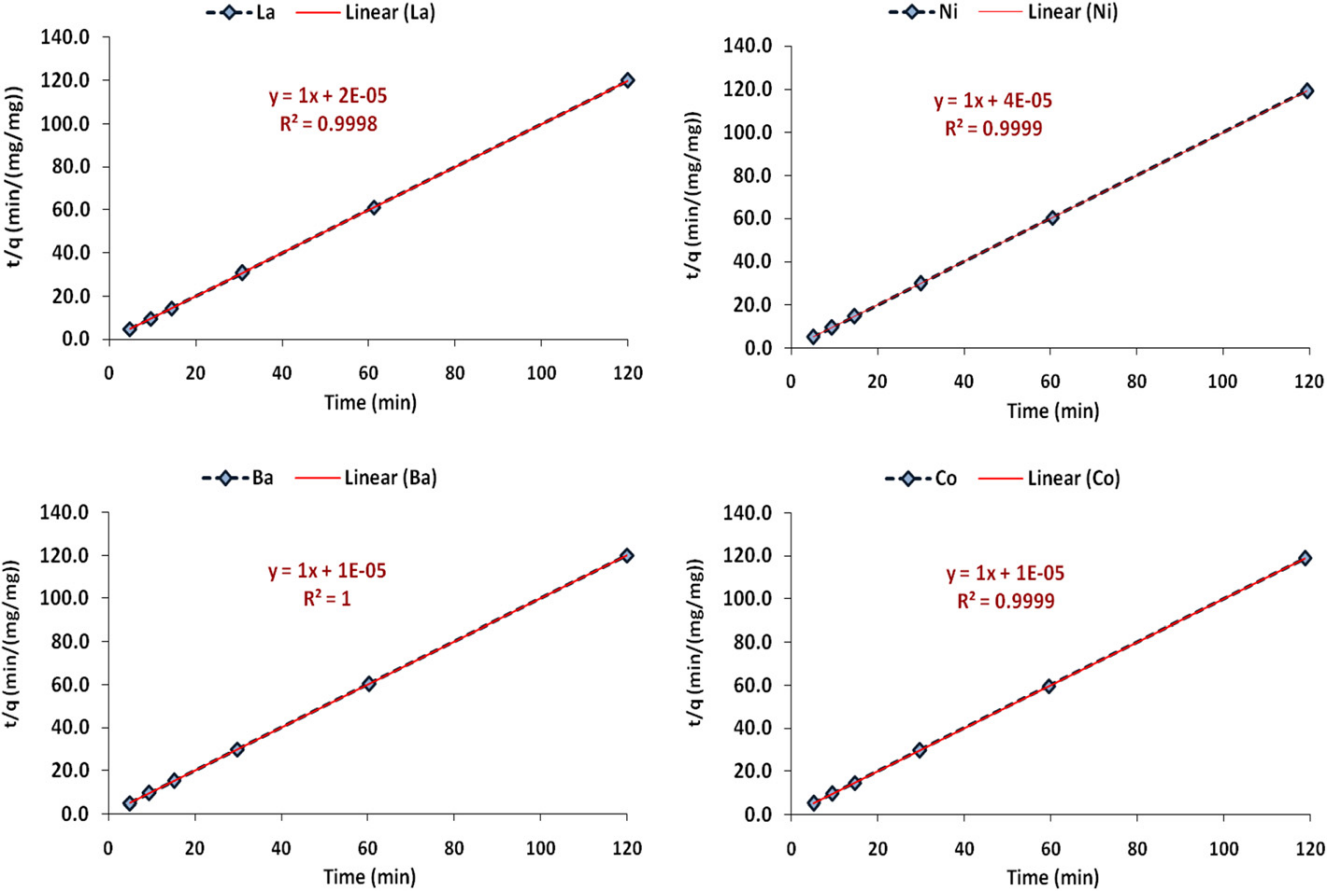
Pseudo-second order kinetic plots for Co (II), Ba (II), La (III) and Ni (II) ions onto Pt/zeolite 4A.

Meanwhile, to design an appropriate adsorption process, one should have sufficient information about the rate at which adsorption occurs. Thus, the rate constants for the adsorption of Co (II), Ba (II), La (III) and Ni (II) ions from aqueous solution on to Pt/zeolite 4A were determined using the pseudo second order equation (Eq. (5)) as it gave the best description of experimental data points:

(5) $$\frac{t}{q}=\frac{1}{K_{2}q_{e}^{2}}+\frac{t}{q_{e}}$$

where K_2_ is the rate of the pseudo second order equation (g/mg.min) and qe is the amount of the metal ions adsorbed per unit gram of adsorbent at equilibrium and time (t) respectively. A plot of t/qt versus t should be linear if the pseudo second order model is obeyed. Figure 9 shows the application of Eq. (5). Linear plots are obtained with R^2^ values of 0.999, 0.999, 0.999 and 1 for Co (II), La (III), Ni (II) and Ba (II), respectively, suggesting that the Pt/zeolite 4A – Co (II), La (III), Ni (II) and Ba (II) ions interaction follows the pseudo second order mechanism. The calculated values of K_2_, qe and R^2^ of each plot are given in Table 3.

**Table 3:** Pseudo-second order kinetic parameters for Co (II), Ba (II), La (III) and Ni (II) ions onto Pt/zeolite 4A

**Metal ions**	**C**_**0**_**(ppm)**	**K**_**2**_**(mg.min)**	**h(mg/g.min)**	**q**_**e**_**(mg/g)**	**R**^**2**^
La	100	50	50	1	0.9998
Ni	100	25	25	1	0.9999
Ba	100	100	100	1	1
Co	100	100	100	1	0.999

## Conclusion

The removal of heavy metals from aqueous solution was carried out in a batch adsorption mode using platinum nanopartcles/Zeolite-4A adsorbent. The platinum nanopartcles/Zeolite-4A exhibited effectiveness in the removal of Co (II), Ba (II), La (III) and Ni (II) ions from aqueous solutions. The removal efficiency was controlled by solution pH, adsorbent concentration and contact times. Adsorption data fitted well with the Freundlich model for Ba (II), while Langmuir isotherm adsorption model having higher R^2^ value for Co (II), La (III) and Ni (II) ions, described the adsorption process better than Freundlich model for the three metals. This novel material opens new door for various usage of the nanomaterials in different fields of application in the wastewater treatment.

## Competing interests

All authors declare that they have no competing interest.

## Authors’ contributions

This study is a part of SM thesis, who prepared the literature survey and performed the experiments. SS participated in the design of the study, data analysis, and manuscript preparation. SJA and MO were advisors of the study. All authors read and approved the final manuscript.

## References

[B1] Ahmadi SJ Sadjadi S Hosseinpour M Adsorption behavior of toxic metal ions on nano-structured CuO granules Separ Sci Technol 2012 47 1063 1069 10.1080/01496395.2011.631675

[B2] Horsfall MJ Spiff AI Effects of temperature on the sorption of Pb^2+^ and Cd^2+^ from aqueous solution by caladium bicolor (wild cocoyam) biomass Electron J Biotechn 2005 8 162 169 10.2225/vol8-issue2-fulltext-4

[B3] Igwe JC Abia AA Maize Cob and Husk as Adsorbents for removal of Cd, Pb and Zn ions from wastewater Phys Sci 2003 2 83 94

[B4] Igwe JC Abia AA A bioseparation process for removing heavy metals from waste water using biosorbents Afr J Biotechnol 2006 5 12 1167 1179

[B5] Esalah OJ Weber ME Vera JH Removal of lead, cadmium and zinc from aqueous solutions by precipitation with sodium di-(n-octyl) phosphinate Can J Chem Eng 2000 78 948 954 10.1002/cjce.5450780512

[B6] Gupta KV Gupta M Sharma S Process development for the removal of lead and chromium from aqueous solutions using red mud-an aluminum industry waste Water Res 2001 35 1125 1234 10.1016/S0043-1354(00)00389-4 11268832

[B7] Kang KC Kim SS Choi JW Kwon SH Sorption of Cu^2+^ and Cd^2+^ onto acid and base pretreated granular activated carbon and activated carbon fiber samples J Ind Eng Chem 2008 14 131 135 10.1016/j.jiec.2007.08.007

[B8] Manohar DM Noeline BF Anirudhan TS Adsorption performance of Al-pillared bentonite clay for the removal of cobalt(II) from aqueous phase Appl Clay Sci 2006 31 194 206 10.1016/j.clay.2005.08.008

[B9] Ewecharoen A Thiravetyan P Wendel E Bertagnolli H Nickel adsorption by sodium polyacrylate-grafted activated carbon J Hazard Mater 2009 171 335 339 10.1016/j.jhazmat.2009.06.008 19576692

[B10] Zhang S Cheng F Tao Z Gao F Chen J Removal of nickel ions from wastewater by Mg(OH)_2_/Mg nanostructures embedded in Al_2_O_3_ membranes J Alloys Compd 2006 426 281 285 10.1016/j.jallcom.2006.01.095

[B11] Chunfeng W Jiansheng LI Xia S Lianjun W Xiuyun S Evaluation of zeolites synthesized from fly ash as potential adsorbents for wastewater containing heavy metals J Environ Sci 2009 21 127 136 10.1016/S1001-0742(09)60022-X 19402411

[B12] Teranish T Hosoe M Tanaka T Miyake M Size control of monodispersed Pt nanoparticles and their 2D organization by electrophoretic deposition J Phys Chem B 1999 103 3818 3827

[B13] Acharya J Sahu JN Mohanty CR Meikap BC Removal of lead (II) from wastewater by activated carbon developed from Tamarind wood by zinc chloride activation J Chem Eng 2009 2009 149 249 262

[B14] Langmuir I The constitution and fundamental properties of solids and liquids. Part I: solids J Am Chem Soc 1916 38 2221 2295 10.1021/ja02268a002

